# Vitamin D supplementation in pregnancy and early infancy in relation to gut microbiota composition and *C. difficile* colonization: implications for viral respiratory infections

**DOI:** 10.1080/19490976.2020.1799734

**Published:** 2020-08-11

**Authors:** Kelsea M. Drall, Catherine J. Field, Andrea M. Haqq, Russell J. de Souza, Hein M. Tun, Nadia P. Morales-Lizcano, Theodore B. Konya, David S. Guttman, Meghan B. Azad, Allan B. Becker, Diana L. Lefebvre, Piush J. Mandhane, Theo J. Moraes, Malcolm R. Sears, Stuart E. Turvey, Padmaja Subbarao, James A. Scott, Anita L Kozyrskyj

**Affiliations:** a Departments of Pediatrics, Obstetrics & Gynecology, University of Alberta, Edmonton, AB, Canada; b Department of Agricultural, Food and Nutritional Science, University of Alberta, Edmonton, AB, Canada; c Department of Health Research Methods, Evidence, and Impact, McMaster University, Hamilton, ON, Canada; d Population Health Research Institute, McMaster University, Hamilton, ON, Canada; e HKU-Pasteur Research Pole, School of Public Health, Hong Kong University, Hong Kong SAR, China; f Dalla Lana School of Public Health, University of Toronto, Toronto, ON, Canada; g Department of Cell & Systems Biology, University of Toronto, Toronto, ON, Canada; h Centre for the Analysis of Genome Evolution & Function, University of Toronto, Toronto, ON, Canada; i Department of Pediatrics & Child Health, Children’s Hospital Research Institute of Manitoba, University of Manitoba, Winnipeg, MB, Canada; j Department of Medicine, McMaster University, Hamilton, ON, Canada; k Department of Pediatrics, Hospital for Sick Children, University of Toronto, Toronto, ON Canada; l Department of Pediatrics, BC Children’s Hospital, University of British Columbia, Vancouver, BC, Canada; m School of Public Health, University of Alberta, Edmonton, Canada

**Keywords:** Vitamin D, supplements, milk, infant, gut microbiota, *C. difficile*, *Megamonas*, *Bilophila*

## Abstract

In Canada and the US, the infant diet is supplemented with vitamin D via supplement drops or formula. Pregnant and nursing mothers often take vitamin D supplements. Since little is known about the impact of this supplementation on infant gut microbiota, we undertook a study to determine the association between maternal and infant vitamin D supplementation, infant gut microbiota composition and *Clostridioides difficile* colonization in 1,157 mother-infant pairs of the CHILD (Canadian Healthy Infant Longitudinal Development) Cohort Study over 2009–2012. Logistic and MaAsLin regression were employed to assess associations between vitamin D supplementation, and *C. difficile* colonization, or other gut microbiota, respectively. Sixty-five percent of infants received a vitamin D supplement. Among all infants, infant vitamin D supplementation was associated with a lower abundance of genus *Megamonas* (q = 0.01) in gut microbiota. Among those exclusively breastfed, maternal prenatal supplementation was associated with lower abundance of *Bilophila* (q = 0.01) and of Lachnospiraceae (q = 0.02) but higher abundance of *Haemophilus* (q = 0.02). There were no differences in microbiota composition with vitamin D supplementation among partially and not breastfed infants. Neither infant nor maternal vitamin D supplementation were associated with *C. difficile* colonization, after adjusting for breastfeeding status and other factors. However, maternal consumption of vitamin-D fortified milk reduced the likelihood of *C. difficile* colonization in infants (adjustedOR: 0.40, 95% CI: 0.19–0.82). The impact of this compositional difference on later childhood health, especially defense against viral respiratory infection, may go beyond the expected effects of vitamin D supplements and remains to be ascertained.

## Introduction

Most infants in North America are supplemented with vitamin D subsequent to recommendations that all breastfed infants receive 400 IU/day, an amount that is also present in commercial infant formulas.^1,2^ However, infant vitamin D supplementation during breastfeeding is not common practice in Australia and several European countries, including Italy and Spain,^3,4^ and compliance rates in Canada and the US are moderate.^5,6^ Vitamin D has shown protective activity against the development of preschool wheeze when provided as a maternal prenatal supplement.^7^ Supplementation of preterm infants with vitamin D has reduced recurrent wheeze in a randomized-controlled trial.^8^ Independent of gestational age and breastfeeding status, infant supplementation before 6-months has shown future benefit in raising serum vitamin D levels and reducing hospital length-of-stay of infants hospitalized for bronchiolitis, a respiratory infection often caused by respiratory syncytial virus (RSV).^9^ Of note, low vitamin D levels has been associated with susceptibility to other viral respiratory infections, including SARS-CoV-2 (COVID-19 disease).^10^ Furthermore, the high rates of food allergy and recurrent wheeze in Australian children have been attributed to vitamin D insufficiency during infancy.^11^

While the immunological roles (both innate and adaptive) of vitamin D have been extensively studied, vitamin D has many physiological roles important for the maintenance of gut microbiota and exclusion of opportunistic microbes. Vitamin D receptors (VDRs) are found on immune cells including T cells, B cells and dendritic cells, but are also highly expressed in intestinal enterocytes where they act as transcription factors for the secretion of antimicrobial peptides and tight junction proteins that maintain intestinal epithelial barrier function.^12,13^ Asystematic review of *in vivo* research has summarized the impact of vitamin D on mammalian gut microbiota^14^ but only included evidence from two studies in human infants. Meanwhile, anewer human study identified prenatal vitamin D supplementation as an important predictor of variance in gut microbial profiles of infants.^15^ In the KOALA birth cohort, *Clostridioides difficile* counts in 1-month old infants were reduced following maternal prenatal supplementation with multivitamins containing vitamin D.^16^ Although asymptomatic *C. difficile* colonization occurs in approximately 30% of young infants, it is less common in breastfed infants and it has been associated with a disrupted gut microbiota composition and later childhood allergic disease. In adults, vitamin D deficiency has also been correlated with *C. difficile* infection, while community incidence of *C. difficile* has been associated with RSV and influenza virus circulation.^17^

Notwithstanding that infant feeding type shapes the infant gut microbiome,^18,19^ attention is shifting to specific micronutrients. If vitamin D influences the gut microbiome, supplementation during breastfeeding may confer additional benefits.^2,20^ The primary objective of this study was to determine the association between maternal perinatal and infant vitamin D supplementation, and infant gut microbiota composition at 3 months of age, including *C. difficile* colonization.

## Results

### Participant characteristics

At an average age of 3.52 months (SD: 0.97 months), 64.7% of our 1,157 infants received a vitamin D_3_ supplement (referred to as vitamin D throughout) in the form of vitamin D drops (Table 1). A high proportion (74.8%) of exclusively breastfed infants received vitamin D supplements. Supplementation was less frequent in partially (67.8%) and exclusively formula fed infants (25.6%) (Table 1). The Manitoba site had the lowest prevalence of infant vitamin D supplementation (43.4% overall, 53.4% in exclusively breastfed infants). Fifteen percent of infants were born to mothers who did not report taking vitamin D supplements or took <400IU/day during pregnancy and lactation, compared to 67% whose mothers reported taking vitamin D supplements (≥400 IU/day) both pre and postnatally.

**Table 1.** Participant characteristics according to vitamin D supplementation and *Clostridioides difficile* colonization in all infants.

	*Maternal supplement intake ≥400 IU/day*						*Infant supplement intake ≥400 IU/day*			*C. difficile colonization*		
*Covariates* Mean, SD	Neither n = 175/ 1,126 (15.54%)	Prenatal n = 108/ 1,126 (9.59%)	Postnatal n = 85/ 1,126 (7.55%)	Both n = 758/ 1,126 (67.32%)		p-value^a^	No n = 409/ 1,157 (35.35%)	Yes n = 748/ 1,157 (64.65%)	p- value^a^	No n = 813/ 1,157 (70.27%)	Yes n = 344/ 1,157 (29.73%)	p- value^a^
Age at stool sample collection, months	3.49 (0.95)	3.52 (0.98)	3.42 (0.96)	3.53 (0.98)		0.787	3.40 (0.94)	3.58 (0.98)	0.002	3.41 (0.84)	3.76 (1.18)	<0.001
Maternal age, years	30.41 (4.97)	31.43 (4.79)	31.68 (4.40)	32.13 (4.54)		<0.001	30.61 (4.61)	32.30 (4.60)	<0.001	31.99 (4.56)	31.03 (4.87)	<0.001
Maternal pre-pregnancy BMI	25.91 (6.16)	25.44 (6.24)	24.55 (5.49)	24.56 (5.15)		0.02	25.81 (6.07)	24.36 (5.05)	<0.001	24.48 (5.02)	25.82 (6.35)	<0.001
Hospital length of stay, days	5.01 (16.60)	4.83 (15.59)	10.56 (27.43)	6.03 (18.84)		0.139	8.89 (24.78)	4.56 (14.69)	<0.001	6.13 (19.32)	5.98 (18.13)	0.905
Introduction to solid foods, months	4.87 (0.97)	5.00 (0.89)	4.98 (0.85)	4.97 (0.97)		0.608	4.80 (0.96)	5.04 (0.86)	<0.001	5.01 (0.89)	4.83 (0.05)	0.002
Birth mode, n (row %)												
Vaginal no IAP	105 (17.07)	55 (8.94)	49 (7.97)	406 (66.02)		0.349	240 (37.91)	393 (62.09)	0.060	466 (73.62)	167 (26.38)	0.001
Vaginal IAP	37 (14.74)	22 (8.76)	19 (7.57)	173 (68.92)			92 (35.38)	168 (64.62)		188 (72.31)	72 (27.69)	
CS Elective	14 (15.91)	12 (13.64)	5 (5.68)	57 (64.77)			25 (28.41)	63 (71.59)		57 (64.77)	31 (35.23)	
CS Emergency	14 (9.33)	17 (11.33)	8 (5.33)	111 (74.00)			43 (27.92)	111 (72.08)		89 (57.79)	65 (42.21)	
Infant Sex, n (row %)												
Female	75 (14.56)	43 (8.35)	40 (7.77)	357 (69.32)		0.436	193 (36.35)	338 (63.65)	0.537	384 (72.32)	147 (27.68)	0.175
Male	100 (16.37)	65 (10.64)	45 (7.36)	401 (65.63)			216 (34.50)	410 (65.50)		429 (68.53)	197 (31.47)	
Feeding mode, n (row %)												
Exclusively breastfed	94 (14.97)	59 (9.39)	52 (8.28)	423 (67.36)		0.354	163 (25.19)	484 (74.81)	<0.001	504 (77.90)	143 (22.10)	<0.001
Partially breastfed	47 (15.26)	29 (9.42)	15 (4.87)	217 (70.45)			101 (32.17)	213 (67.83)		204 (64.97)	110 (35.03)	
Exclusively formula fed	33 (17.46)	20 (10.58)	18 (9.52)	118 (62.43)			145 (74.36)	50 (25.64)		104 (53.33)	91 (46.67)	
Infant antibiotics, n (row %)												
No	169 (15.43)	103 (9.41)	82 (7.49)	741 (67.67)		0.335	400 (35.56)	725 (64.44)	0.456	792 (70.40)	333 (29.60)	0.560
Yes	6 (19.35)	5 (16.13)	3 (9.68)	17 (54.84)			9 (28.12)	23 (71.88)		21 (65.62)	11 (34.38)	
Household income, n (row %)												
≤ $39999	17 (19.54)	10 (11.49)	12 (13.79)	48 (55.17)		0.027	50 (53.19)	44 (46.81)	<0.001	64 (68.09)	30 (31.91)	0.940
$40,000 to $79,999	58 (19.73)	32 (10.88)	19 (6.46)	185 (62.93)			119 (40.07)	178 (59.93)		213 (71.72)	84 (28.28)	
$80,000 to $99,999	20 (12.50)	19 (11.88)	9 (5.62)	112 (70.00)			54 (33.54)	107 (66.46)		115 (71.43)	46 (28.57)	
≥ $100,000	62 (13.30)	33 (7.08)	38 (8.15)	333 (71.46)			127 (27.14)	341 (72.86)		332 (70.94)	136 (29.06)	
Preferred not to answer	15 (14.29)	14 (13.33)	7 (6.67)	69 (65.71)			47 (44.76)	58 (55.24)		72 (68.57)	33 (31.43)	
Maternal ethnicity, n (row %)												
Caucasian	128 (15.17)	81 (9.60)	68 (8.06)		567 (67.18)	0.008	309 (35.97)	550 (64.03)	0.075	589 (68.57)	270 (31.43)	0.002
Asian	18 (10.84)	14 (8.43)	7 (4.22)		127 (76.51)		47 (27.98)	121 (72.02)		137 (81.55)	31 (18.45)	
Other	29 (25.22)	13 (11.30)	10 (8.70)		63 (54.78)		48 (39.67)	73 (60.33)		82 (67.77)	39 (32.23)	
Maternal Depression, n (row %)												
None	103 (13.62)	63 (8.33)	60 (7.94)		530 (70.11)	0.068	254 (33.12)	513 (66.88)	0.110	550 (71.71)	217 (28.29)	0.155
Prenatal	33 (22.00)	16 (10.67)	10 (6.67)		91 (60.67)		62 (39.74)	94 (60.26)		98 (62.82)	58 (37.18)	
Postnatal	18 (17.14)	11 (10.48)	7 (6.67)		69 (65.71)		43 (37.39)	72 (62.61)		83 (72.17)	32 (27.83)	
Both	21 (18.26)	18 (15.65)	8 (6.96)		68 (59.13)		50 (42.74)	67 (57.26)		80 (68.38)	37 (31.62)	
Season of birth, n (row %)												
October through March (low UV)	82 (13.97)	62 (10.56)	51 (8.69)		392 (66.78)	0.151	206 (37.39)	345 (62.61)	0.176	436 (71.95)	170 (28.05)	0.198
April through September (high UV)	93 (17.25)	46 (8.53)	34 (6.31)		366 (67.90)		203 (33.50)	403 (66.50)		377 (68.42)	174 (31.58)	
Maternal prenatal milk consumption, n (row %)												
1 or less cups a day	9 (11.69)	4 (5.19)	5 (6.49)		59 (76.62)	0.654	25 (31.65)	54 (68.35)	0.421	52 (65.82)	27 (34.18)	0.014
2 cups a day	52 (15.16)	32 (9.33)	27 (7.87)		232 (67.64)		131 (37.75)	216 (62.25)		227 (65.42)	120 (34.58)	
3 or cups a day	103 (15.85)	69 (10.62)	43 (6.62)		435 (66.92)		223 (34.15)	430 (65.85)		482 (73.81)	171 (26.19)	
Pets in the home, n (row %)												
No	91 (14.99)	58 (9.56)	47 (7.74)		411 (67.71)	0.960	211 (34.03)	409 (65.97)	0.354	455 (73.39)	165 (26.61)	0.014
Yes	83 (16.12)	49 (9.51)	38 (7.38)		345 (66.99)		195 (36.65)	337 (63.35)		355 (66.73)	177 (33.27)	
Study center, n (row%)												
Edmonton	41 (15.30)	26 (9.70)	19 (7.09)		182 (67.01)	0.022	59 (21.38)	217 (78.62)	<0.001	161 (58.33)	115 (41.67)	<0.001
Vancouver	42 (11.23)	31 (8.29)	25 (6.68)		276 (73.80)		66 (17.41)	313 (82.59)		292 (77.04)	87 (22.96)	
Manitoba	92 (19.01)	51 (10.54)	41 (8.47)		300 (67.32)		284 (56.57)	218 (43.43)		360 (71.71)	142 (28.29)	
Maternal intake, n (row%)												
None or below recommendation							88 (50.29)	87 (49.71)	<0.001	125 (71.43)	50 (28.57)	0.996
Prenatal							32 (29.63)	76 (70.37)		76 (70.37)	32 (29.63)	
Postnatal							34 (40.00)	51 (60.00)		60 (70.59)	25 (29.41)	
Both							241 (31.79)	517 (68.21)		533 (70.32)	225 (29.68)	
Infant intake, n (row%)												
No	88 (22.28)	32 (8.10)	34 (8.61)		241 (61.01)	<0.001				272 (66.50)	137 (33.50)	0.043
Yes	87 (11.90)	76 (10.40)	51 (6.98)		517 (70.73)					541 (72.33)	207 (27.67)	
*C. difficile* colonization, n (row%)												
No	125 (15.74)	76 (9.57)	60 (7.56)		533 (67.13)	0.996	272 (33.46)	541 (66.54)	0.043			
Yes	50 (15.06)	32 (9.64)	25 (7.53)		225 (67.77)		137 (39.83)	207 (60.17)				

^a^P values **bolded** if p ≤ 0.05, and calculated using Fisher’s exact tests, t-tests and ANOVA (analysis of variance) in Stata (version 13.0)

Abbrev. SD: standard deviation, IAP: intrapartum antibiotic prophylaxis, CS: cesarean section, UV: ultraviolet light

Overall, 29.7% of infants were colonized with *C. difficile*, but less among exclusively breastfed infants (22.1%, Table 1). Among those infants who were colonized with *C. difficile*, mothers were usually younger, had a higher BMI, and were less frequently of Asian ethnicity (Table 1). Asmaller proportion of infants colonized with *C. difficile* were born to mothers who consumed at least 3 cups of fortified milk a day, were more frequently living in homes with furry pets, born in Edmonton and less likely to receive vitamin D drops.

### Maternal and infant vitamin D supplement intake is not associated with infant C. difficile colonization

In all infants, direct vitamin D supplementation (OR: 0.76, 95% CI: 0.59–0.99, *p* = .04), as well as postnatal supplementation (≥ 2 sources of vitamin D, (OR: 0.67, 95% CI: 0.45–0.98, *p* = .04), were associated with lower odds of *C. difficile* colonization compared to the absence of supplementation; however, these associations were lost upon adjustment for covariates, notably infant feeding mode. Prenatal maternal vitamin D supplementation was not associated with infant *C. difficile* colonization in crude or adjusted models.

**Figure 1.**
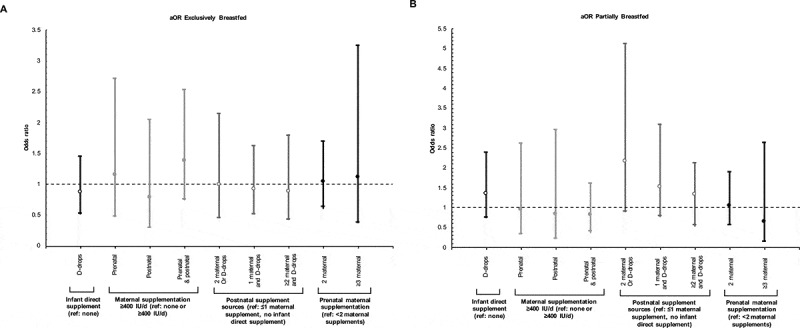
*C. difficile* colonization according to maternal or infant vitamin D supplementation in the perinatal period.

Within exclusively breastfed infants, the crude odds ratios for *C. difficile* colonization were not statistically significant with infant vitamin D supplementation (OR: 0.87, 95% CI: 0.57–1.32, *p* = .585) nor with maternal supplementation (Table S1). These null associations were unchanged following adjustment for birth mode, maternal milk consumption, household pets, study center and infant age at sample collection (Figure 1a, Table S1). No significant interaction terms were discovered between variables. Asensitivity analysis was conducted to ensure that results were not affected by site variations in infant supplementation. No deviations from the main findings were noted for associations between vitamin D supplementation and infant *C. difficile* colonization after the Manitoba site was excluded (data not shown).

(a): Exclusively breastfed infants (N = 647), (b) Partially breastfed infants (N = 314). Exclusively formula fed infants (excluded non-direct postnatal, ie. maternal sources during lactation from this model, presented in Figure 3). Adjusted for maternal milk consumption during pregnancy, household pets, age at stool sample collection, study center and other supplement categories; D-drop use adjusted for maternal D supplementation (pre/post); post-natal supplements adjusted for pre-natal supplement use; and pre-natal supplement use adjusted for post-natal supplement use (maternal and infant). Adjusted odds ratios (aOR) and 95% confidence intervals (error bars) calculated using logistic regression in Stata (version 13.0).

Independent of infant or maternal prenatal vitamin D supplementation, prenatal maternal milk consumption greater than or equal to 3 cups per day, compared to 1 or less cups per day, was significantly associated with lower odds (aOR: 0.40, 95% CI: 0.19–0.82, *p* = .01, Table S2) of *C. difficile* colonization in exclusively breastfed infants. In this final adjusted model, *C. difficile* colonization was twice more likely in infants born via elective cesarean and in the presence of household pets (Table S2). No associations between vitamin D supplementation and *C. difficile* colonization were observed (summary Table 2) within partially (Figure 1b) or exclusively formula fed infants (Figure S1).

**Table 2.** **Summary of study results**. Prevalence of vitamin D supplementation/sources and the association with *C. difficile* colonization and gut microbiota composition.

Exposure group (sources of early life vitamin D)	Stratified group	Prevalence of exposure	Association with *C. difficile* colonization	Association with other gut microbiota (family and genus level)
Infant vitamin D supplementation (i.e. vitamin D-drops, ref: none)	All (crude)	64%	24% lower odds	Not tested
	All (adjusted)	64%	_	↓ Veillonellaceae, Megamonas*
	Exclusively breastfed	75%	_	_
	Partially breastfed	68%	_	_
	Exclusively formula fed	26%	_	_
Maternal prenatal and postnatal vitamin D supplementation ≥400 IU/day (ref: <400 IU/day)	All (crude)	84%	33% lower odds	Not tested
	All (adjusted)	84%	_	_
	Exclusively breastfed	85%	_	↓ Desulfovibrionaceae, Bilophila** ↓ Lachnospiraceae, Other** ↑ Pasteurellaceae, Haemophilus**
	Partially breastfed	85%	_	_
	Exclusively formula fed	83%	_	_


*Adjusted for breastfeeding status, maternal pre-post vitamin D supplementation and mode of delivery and all other microbiota (calculated using MaAsLin)

**Adjusted for mode of delivery, infant vitamin D supplementation and all other microbiota (calculated using MaAsLin)

### Vitamin D supplementation is associated with other compositional changes in infant gut microbiota

Following adjustment for birth mode and feeding mode, microbiota of the genus *Megamonas* (Veillonellaceae family) were of significantly lower abundance in all infants supplemented with vitamin D (q = 0.01, Table 3). Furthermore, *Peptostreptococcus* (Peptostreptococcaceae family) were lower in supplemented infants and *Eubacterium* (Eubacteriaceae family) were depleted in infants whose mothers took a pre and postnatal vitamin D supplement (>400IU/day); however statistical significance for these two genera was lost upon FDR correction (Table 3).

**Table 3.** **Gut microbiota composition in all study infants**. Multivariate linear regression (MaAsLin) predicting arcsine square root transformed relative abundances of microbiota in all study infants (N = 1,157), exclusively breastfed infants (N = 647), partially breastfed (N = 314) and exclusively formula fed (N = 195) according to postnatal maternal and infant vitamin D supplementation practices.

Postnatal Infant Vitamin D Supplementation (ref: none)		Coef^a^	SE	N	Non zero N	p-value	q-value^b^
Firmicutes|Clostridia|Clostridiales|Veillonellaceae| *Megamonas*		−0.0049	0.0016	1157	45	0.002	0.012
Firmicutes|Clostridia|Clostridiales|Peptostreptococcaceae *Peptostreptococcus*		−0.0017	0.0008	1157	54	0.038	0.173
EBF infants	Proteobacteria|Deltaproteobacteria| Desulfovibrionales|Desulfovibrionaceae *Bilophila*	−0.0087	0.0029	647	95	0.0029	0.169
PBF infants	Proteobacteria|Gammaproteobacteria| Pasteurellales|Pasteurellaceae *Haemophilus*	0.0098	0.0033	314	183	0.003	0.052
	Firmicutes|Clostridia|Clostridiales| Ruminococcaceae| *Ruminococcus*	−0.0083	0.0029	314	152	0.004	0.073
	Firmicutes|Clostridia|Clostridiales| Veillonellaceae| *Megamonas*	−0.0010	0.0040	314	7	0.014	0.196
EFF	Bacteroidetes|Bacteroidia|Bacteroidales| Odoribacteraceae| *Butyricimonas*	0.0100	0.0036	195	11	0.006	0.103
Maternal Pre and Postnatal Vitamin D Supplementation (ref: none or <400IU/day)		Coef^a^	SE	N	Non zero N	p-value	q-value^b^
Firmicutes|Clostridia|Clostridiales|Eubacteriaceae| *Eubacterium*		−0.0004	0.0002	1157	38	0.028	0.127
EBF infants	Proteobacteria|Deltaproteobacteria| Desulfovibrionales| Desulfovibrionaceae *Bilophila*	−0.0038	0.0011	647	95	0.0007	0.009
	Firmicutes|Clostridia|Clostridiales| Lachnospiraceae*|* Other	−0.0010	0.0003	647	229	0.0015	0.018
	Proteobacteria|Gammaproteobacteria| Pasteurellales|Pasteurellaceae *Haemophilus*	0.0058	0.0018	647	520	0.0018	0.021
PBF infants	Firmicutes|Erysipelotrichi|Erysipelotrichales |Erysipelotrichaceae Unclassified	−0.0027	0.0010	314	155	0.005	0.089

^a^Coef: Coefficient: Arcsine square root transformed regression beta, calculated using MaAsLin

^b^q-values FDR corrected, adjusted for feeding mode (in non-stratified analyses) and birth mode

EBF: Exclusively Breastfed, PBF: Partially Breastfed, EFF: Exclusively Formula Fed

Exclusively breastfed infants born to mothers who were taking ≥400IU/day both pre and postnatally exhibited a lower relative abundance of Proteobacteria, specifically those of the genus *Bilophila* (q = 0.01, Table 3). Furthermore, bacteria belonging to the Lachnospiraceae family (q = 0.02) were depleted and microbes belonging to the Pasteurellaceae family (*Haemophilus* spp., q = 0.02) were enriched compared to infants nursed by mothers taking <400IU/day (Table 3). The same modeling procedure was followed for partially and exclusively formula fed infants but none of the associations survived FDR correction (summary Table 2).

## Discussion

With the aim to address a gap in emerging discussions on vitamin D and intestinal homeostasis in infants,^14^ we undertook this gut microbiome study in a general population of 1,157 Canadian mothers and infants. Three-quarters of breastfed infants were supplemented with vitamin D drops and 85% of mothers took vitamin supplements containing ≥400 IU of vitamin D daily during pregnancy and postnatally. At 3–4 months of age, *C. difficile* colonization of the infant gut was less prevalent with infant vitamin D supplementation but this was explained by the extent of breastfeeding, a strong deterrent of *C. difficile*.^21^ Within exclusively breastfed infants, neither infant nor maternal vitamin D supplementation were related to *C. difficile* colonization status. Whereas *C. difficile* counts were reportedly lower in colonized, breastfed infants of mothers with prenatal vitamin D intake ≥400 IU/day in the KOALA study, infant *C. difficile* colonization rates in this cohort did not differ by infant vitamin D supplement intake.^16^ Our findings agree with those of the KOALA study regarding direct vitamin D supplementation of breastfed infants, for which there were no associations with *C. difficile* colonization of their gut microbiota.^16^

However, we found direct vitamin D supplementation of infants to be associated with a lower abundance of *Megamonas* in gut microbiota. This association was found in all study infants and was independent of feeding mode. While little is known about *Megamonas* in infancy, this genus was enriched in the gut microbiota of male infants born to mothers with prenatal asthma in a previous publication from the CHILD cohort.^22^ In addition to childhood asthma, maternal history of asthma is a risk factor for greater severity of viral bronchiolitis in offspring.^23^ *Megamonas* has recently been reported to be more abundant in the gut microbiota of adult males with higher testosterone levels^24^ and the role of this sex steroid is currently being scrutinized in asthma pathogenesis.^25^ In view of reported benefits of the vitamin D supplementation of high risk mothers and of infant populations where supplementation is not the norm,^7,11^ the *Megamonas* genus of Veillonellaceae may be a possible link between vitamin D and asthma or viral respiratory infection that merits further examination. It would also further support recommendations for the supplementation of formula-fed infants. Vitamin D plays a crucial role in both innate immunity, via toll-like receptor signaling of macrophages in response to pathogens, and adaptive immunity, by inhibiting proliferation of T cells and secretion of inflammatory cytokines.^13,26^ Further, there is emerging evidence for its role in the lung microbiome and gut-lung axis.^27^

We also found associations with maternal pre and postnatal vitamin D supplementation (≥400IU/day) in exclusively breastfed infants, namely a lower relative abundance of *Bilophila* spp. *Bilophila* have been linked to inflammation and colitis in mice,^28,29^ and colic in infants.^30^ Genus *Bilophilia* produce secondary bile acids, ligands for VDRs, and levels of these microbiota are elevated in the presence of taurine or bile.^31–33^ Vitamin D receptors are highly expressed in the proximal colon and involved in the production of defensins, cathelicidins, claudins and zonulin occludens, important to gut barrier integrity.^12,34,35^ Newborns conjugate bile acids with taurine but have the capacity to utilize glycine, especially when fed formula.^31–33,36^ This may explain why *Bilophila* species were uniquely altered among exclusively breastfed infants. Maternal supplementation with vitamin D was also associated with depletion of Lachnospiraceae but enhancement of *Haemophilus* spp, both of which are reported to be altered in mammalian studies of vitamin D.^14^ The reduction in abundance of *Bilophila* and other changes to infant gut microbiota following maternal vitamin D supplementation points to pathways involving production of secondary bile acids.

Interestingly, maternal prenatal consumption of 3 or more cups of fortified milk per day reduced the likelihood of *C. difficile* colonization in exclusively breastfed infants by 60%, even after adjustment for infant vitamin D supplementation, maternal vitamin D supplementation and other covariates (aOR: 0.40, 95% CI: 0.19–0.82, *p* = .01, Table S2). Transitioning to cow’s milk after exclusive breastfeeding seems to have an inhibitory influence on *C. difficile* colonization in infants,^37^ whereas *C. difficile* infection is more common in children with cow’s milk intolerance.^38^ Furthermore, greater maternal dietary intake of vitamin D during pregnancy has been reported to reduce risk of cow’s milk allergy in offspring.^39^ Our findings suggest a putative role of vitamin D fortified-milk consumption during pregnancy but maternal milk consumption may be equally correlated with other maternal dietary patterns^40-42^ or lifestyle factors (i.e. cleaning product use^43^) that influence the composition of infant gut microbiota.

### Study limitations

An important limitation of this work is that we could not distinguish between those with darker skin pigmentation and other ethnicities, beyond Caucasian and Asian. These populations have been shown to produce less subcutaneous vitamin D, making them at greater risk of low vitamin D.^44^ We also observed study site differences specifically that the prevalence of Manitoban infant supplementation was lower, in just over 50% of exclusively breastfed infants. According to the sensitivity analysis we conducted, study findings were unchanged if the Manitoba site was excluded.

Finally, we did not have access to maternal or infant serum vitamin D levels. However, for microbiome research, intestinal levels are likely more important than serum levels and reference values for intestinal vitamin D concentrations have yet to be determined. Instead, this study relied on self-report questionnaires for reporting of vitamin D supplementation. Questionnaire response categories did not allow us to explore potential differences within the group of mothers taking more than 400IU/day. Future studies would benefit from a more specific nutrition/supplement question, such as the one administered in the Alberta Pregnancy Outcomes and Nutrition (APrON) study, which was pilot-tested to ensure efficient and detailed collection of vitamin intake and dosing in the Canadian context.^45^ However, this limitation is not likely to have a large effect on our findings as the APrON study found that the average vitamin D intake during pregnancy from supplements and diet combined was 600 IU/day.^46^

Ultimately, this study found evidence of an association between maternal vitamin D supplementation with the gut microbiota composition of all study infants, notably a lower abundance of *Megamonas*, with its potential implications for host defense against viral respiratory infections. In exclusively breastfed infants, we found evidence of an association between direct vitamin D supplementation and lower abundance of *Bilophila* and members of the Lachnospiraceae, and a higher abundance of *Haemophilus* at 3-months of age. Yet, vitamin D supplementation did not appear to be associated with *C. difficile* colonization in any of the feeding groups. It is essential to confirm our findings to fully comprehend the relationship between vitamin D and the gut microbiota of infants, and to understand how current standards of care around vitamin D supplementation support healthy development.

## Materials and methods

### Study population and design

This observational study included 1,157 families participating in the CHILD Cohort Study.^47^ Mothers were recruited during their second trimester of pregnancy between January 2009 and December 2012 from the Vancouver, Edmonton or Manitoba sites (inclusion and exclusion criteria outlined at www.childstudy.ca). All study infants provided a fecal sample and data on breastfeeding status and infant vitamin D supplement intake (Figure S2). Mothers provided informed consent upon enrollment and the Human Research Ethics Boards at the University of Manitoba, University of Alberta, and University of British Columbia approved this study.

### qPCR for C. difficile detection and 16S sequencing for fecal microbiota analysis

Fecal samples were collected at 3–4 months of age using a standardized protocol during a planned home visit. Methods of sample collection, DNA extraction and amplification, 16S ribosomal RNA sequencing, and microbial taxonomic classification are described elsewhere.^18^

Briefly, collected samples were aliquoted stored at −80°C until analyzed. DNA extraction was performed using 80–200 mg of frozen sample using the QIAamp DNA Stool Mini kit (Qiagen Inc, Valencia CA). Bacterial 16S rRNA genes were amplified at the hypervariable V4 region and sequenced using the Illumina MiSeq platform (San Diego, CA). Sequences were clustered with USEARCH (version 6.1)^48^ at >97% similarity against the GREENGENES reference database (version 13.8) for taxonomic classification in QIIME 1.8 and excluded if <60% similarity. Taxon relative abundance was the outcome variable for the microbiota composition analysis. a specific 16S primer was used for targeted amplification and quantification of *C. difficile*, as described elsewhere.^21^ Multiplex assays were prepared using the QuantiNova Multiplex PCR Kit (QIAGEN) with appropriate primers and probes. Each qPCR reaction cycle consisted of an initial denaturation for 2 min at 95.0◦C, 40 cycles of denaturation for 5 s at 95◦C and annealing/extension/reading for 20 s at 60◦C and was performed on the MiniOpticonTM Real-Time PCR System (Bio-Rad, Hercules, CA, USA). The outcome variable for *C. difficile* presence was fecal colonization status, yes/no.

### Vitamin D exposure variables

Maternal vitamin D supplementation from various sources (prenatal vitamins, multivitamins or vitamin D supplements, including dose and frequency of intake) was collected in questionnaires during pregnancy (Figure S3) and 3-months postpartum (Figure S4). Mothers were asked about infant supplementation with vitamin D_3_ drops (referred to as vitamin D throughout) during the first three months (Figure S4). Maternal and infant supplementation variables were created following this algorithm:

***i. Maternal prenatal vitamin D intake***: a 3-category exposure variable was created from the relevant questionnaire information: 1) One supplement ***or*** no supplements containing vitamin D, 2) Two supplements containing vitamin D and 3) Three or more supplements containing vitamin D.

***ii. Postnatal vitamin D intake***: a 4-category exposure variable was created from the relevant questionnaire information: 1) Low (i.e. one maternal source, no infant direct) *or* no vitamin D from supplements, 2) Two maternal supplements with vitamin D *or* infant direct vitamin D, 3) Infant direct *and* one maternal supplement, and 4) Two or more maternal supplements *and* infant direct vitamin D.

***iii. Maternal perinatal intake based on the dosing information.*** Based on current Dietary Reference Intakes for vitamin D supplementation of 400 IU/day, and a recommended dietary allowance of 600 IU/day during pregnancy and breastfeeding,^44,49^ a final 4 category variable was created: 1) No maternal vitamin D or less than 400 IU/day, 2) Prenatal only maternal vitamin D supplementation ≥400 IU/day, 3) Postnatal only maternal vitamin D supplementation ≥400 IU/day and 4) Prenatal and Postnatal supplementation ≥400 IU/day.

Furthermore, mothers reported their milk intake in a food frequency questionnaire administered during pregnancy as milk/fortified substitute beverage consumption (1 cup), milk/fortified substitute use on cereal (1/2 cup) and milk/fortified substitute in tea/coffee (1 Tbsp). To measure dietary sources of vitamin D through fortified milk and/or plant-based alternatives, a 3-category variable was created: 1) 1 or fewer cups/day, 2) 2 cups/day, 3) 3 or more cups/day.

### Other covariate data

Data from study questionnaires or medical charts were obtained and used to create the following covariates: season of birth (low UVB season, October – March); high UVB season, April – September),^50^ maternal pre-pregnancy age and body-mass-index (BMI), infant age at stool collection, hospital length of stay at birth, mode of delivery, infant sex, feeding mode at stool collection (exclusively breastfed [no non-human milk, juices, formula or solids], partially breastfed or exclusively formula fed), antibiotics use, household income, ethnicity, maternal depressive symptoms, pets in the home and study center.

### Statistical analysis

All descriptive (Fisher’s exact tests, t-tests, ANOVA) and regression tests were completed using Stata (version 13.0) statistical software and the online Galaxy platform (version 1.0.1). Logistic regression models were used to determine the association between vitamin D supplement use and *C. difficile* colonization and were built using purposeful selection of covariates.^51^ Models^52^ were run in all infants, then stratified by feeding mode due to the strong association between breastfeeding and infant vitamin D supplementation. One infant did not have data on feeding mode (stratified analyses, N = 1,156). Microbial taxon abundance was compared using Multivariate Association with Linear Models (MaAsLin), which was adjusted for covariates and subjected to false discovery rate (FDR) correction with q ≤ 0.05.

## Supplementary Material

Supplemental Material

## Data Availability

The data and analysis code that support the findings of this study can be made available from the corresponding author and CHILD Cohort Study coordinators upon reasonable request. These data, including study participant data, are securely stored in the https://childdb.ca database.
