# Supplementary material for: Vitamin D supplementation in pregnancy and early infancy in relation to gut microbiota composition and C. difficile colonization: implications for viral respiratory infections
Source: Gut Microbes. 2020 Aug 11;12(1):1799734. doi: 10.1080/19490976.2020.1799734 (PMC7524344; doi:10.1080/19490976.2020.1799734)
Supplement: Supplemental Material [file KGMI_A_1799734_SM4631.docx]

**Online Supplemental Material**

**Figure S1:** ***C. difficile* colonization among formula fed infants (N=195) according to maternal prenatal or infant vitamin D supplementation.** Adjusted for birth mode, maternal milk consumption during pregnancy, household pets, age at stool sample collection, study centre and other supplement categories; D-drop use adjusted for prenatal maternal vitamin D supplementation; pre-natal supplement use adjusted for infant direct supplement use. Adjusted odds ratios (aOR) and 95% confidence intervals (error bars) calculated using logistic regression in Stata (version 13.0).

**Figure S2: Participant and Data Availability Flow Chart.**

**Figure S3: Prenatal Maternal Supplementation Questionnaire (relevant questions only).**


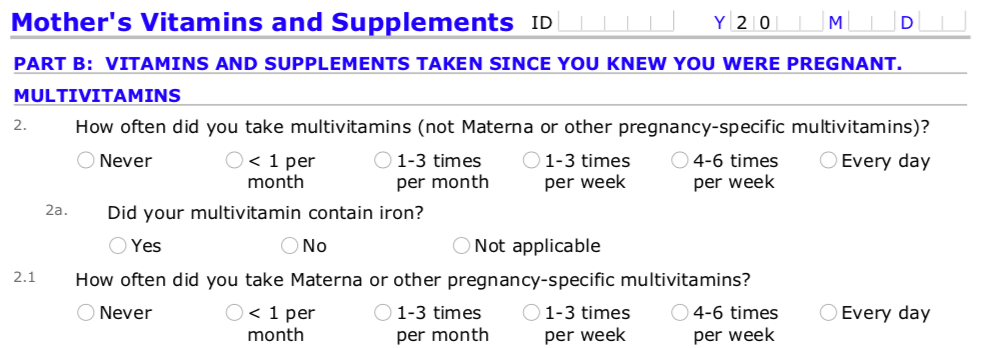


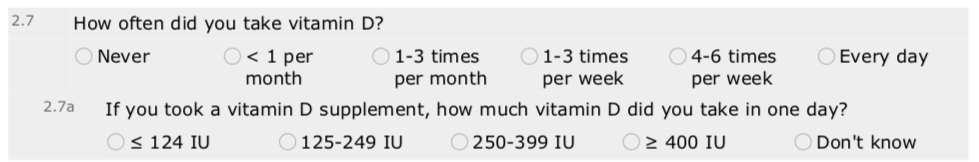


**Figure S4: Three Month Maternal Supplementation Questionnaire and Infant Nutrition Questionnaire (relevant questions only).**


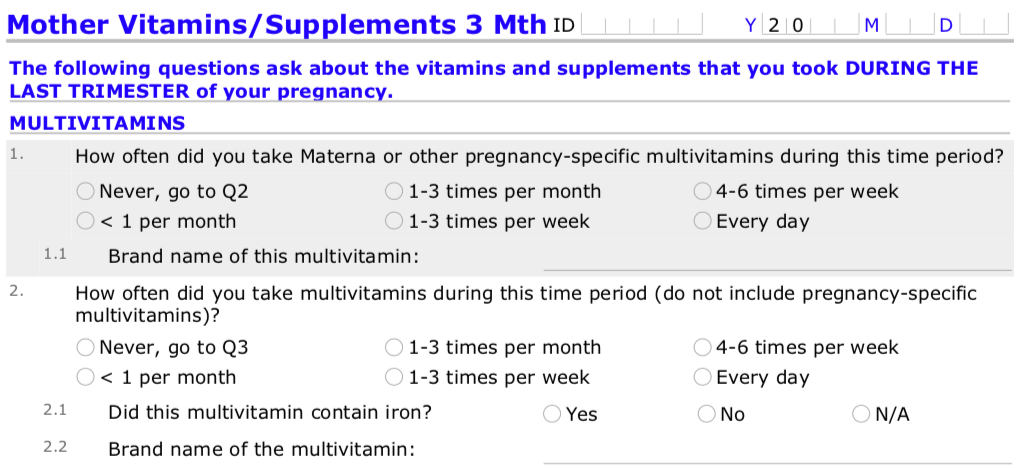


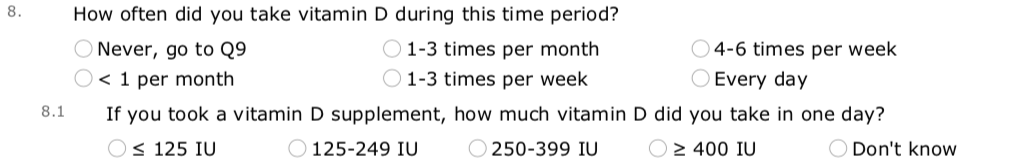


**
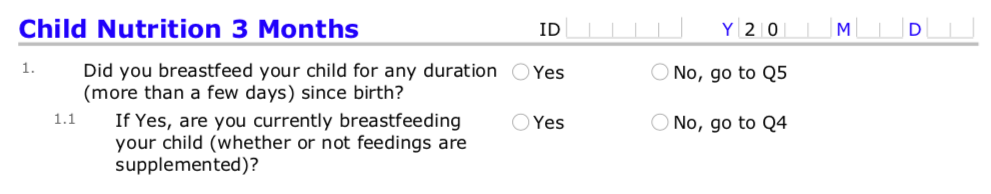
**

**
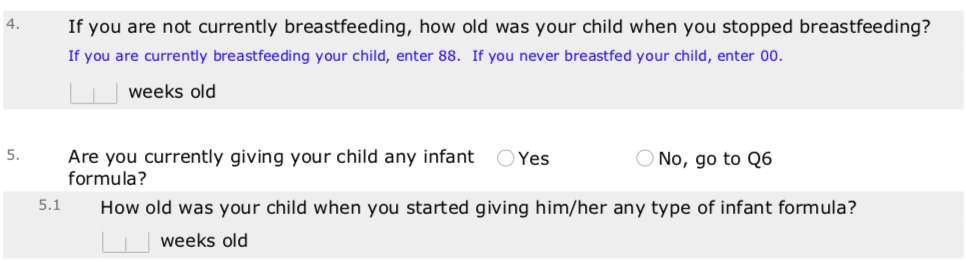
**

**
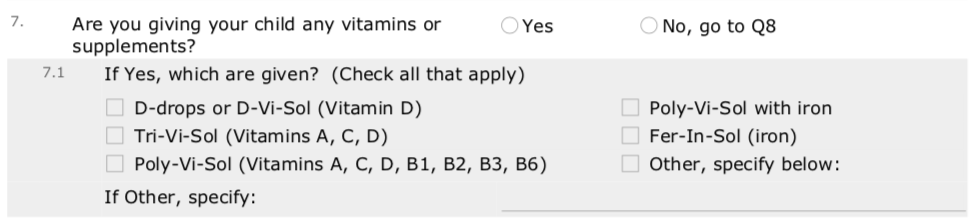
**

**Table S1: Purposeful regression modeling for predicting *C. difficile colonization* exclusively breastfed infants.** Logistic regression analyses of vitamin D supplementation and *C. difficile* colonization in exclusively breastfed infants (N=647). Odds ratios (OR) calculated using Stata (version 13.0).

| Ref: *C. difficile* absent | OR^a^ (95% CI)  infant direct supplementation and *C. difficile* colonization | OR^a^ (95% CI) maternal  prenatal only supplementation (≥400 IU/d) and *C. difficile* colonization | OR^a^ (95% CI) maternal  postnatal only supplementation (≥400 IU/d) and *C. difficile* colonization | OR^a^ (95% CI) maternal pre  and postnatal supplementation (≥400 IU/d) and *C. difficile* colonization |
| --- | --- | --- | --- | --- |
| Crude | 0.87  (0.57 - 1.32) | 1.01  (0.45 - 2.26) | 0.72  (0.29 - 1.78) | 1.17  (0.68 - 2.04) |
| Adjusted for child age  at sample collection | 0.79  (0.52 - 1.21) | 1.02  (0.45 - 2.29) | 0.77  (0.31 - 1.91) | 1.19  (0.68 - 2.07) |
| Adjusted for maternal age | 0.95  (0.62 - 1.46) | 1.06  (0.47 - 2.38) | 0.78  (0.31 - 1.94) | 1.27  (0.73 - 2.22) |
| Adjusted for pre-pregnancy maternal BMI | 1.00  (0.64 - 1.54) | 1.02  (0.43 - 2.38) | 0.67  (0.26 - 1.77) | 1.33  (0.74 - 2.37) |
| Adjusted for birth mode | 0.84 (  0.55 - 1.28) | 1.00  (0.44 - 2.26) | 0.72  (0.29 - 1.79) | 1.14  (0.65 - 1.99) |
| Adjusted for maternal ethnicity | 0.92  (0.60 - 1.41) | 1.00  (0.44 - 2.26) | 0.69  (0.28 - 1.72) | 1.21  (0.69 - 2.11) |
| Adjusted for season of birth | 0.87  (0.57 - 1.33) | 1.01  (0.45 - 2.28) | 0.72  (0.29 - 1.78) | 1.18  (0.68 - 2.04) |
| Adjusted for maternal milk consumption | 1.00 (0.63 - 1.57) | 0.99  (0.42 - 2.30) | 0.85  (0.33 - 2.16) | 1.19  (0.67 - 2.13) |
| Adjusted for pets in the home | 0.89  (0.58 - 1.37) | 1.15  (0.50 - 2.61) | 0.75  (0.30 - 1.88) | 1.30  (0.74 - 2.29) |
| Adjusted for study centre | 0.81  (0.51 - 1.30) | 0.96  (0.42 - 2.18) | 0.66  (0.26 - 1.67) | 1.17  (0.66 - 2.05) |
| Adjusted for maternal vitamin D supplement intake | 0.91  (0.57 - 1.43) | N/A | N/A | N/A |
| Adjusted for infant vitamin D supplement intake | N/A | 1.03  (0.46 - 2.35) | 0.73  (0.29 - 1.80) | 1.19  (0.68 - 2.10) |
| Adjusted for all the above | 1.00  (0.57 - 1.76) | 1.14  (0.43 - 3.01) | 0.94  (0.33 - 2.68) | 1.68  (0.85 - 3.33) |
| Final purposeful model (includes birth mode, milk consumption, pets, study centre and child age at stool sample collection) | 0.86  (0.51 - 1.46) | 1.15  (0.46 - 2.87) | 0.95  (0.36 - 2.54) | 1.43  (0.75 - 2.70) |
| *^a^Odds ratios (OR) and 95% confidence intervals (CI) calculated using logistic regression*  *Abbrev. OR (odds ratio), CI (confidence interval)* | | | | |

**Table S2: Final purposeful regression model for predicting *C. difficile* colonization in exclusively breastfed infants.** Adjusted odds ratios (aOR) calculated using logistic regression in Stata (version 13.0).

|  | aOR for *C. difficile* colonization | p-value^a^ | 95% CI |
| --- | --- | --- | --- |
| Ref: no *C. difficile* colonization |  |  |  |
| Infant direct supplementation (ref: none) | 0.86 | 0.585 | 0.51 -1.46 |
| Maternal prenatal only supplementation ≥400 IU/day (ref: <400 IU/d or none) | 1.15 | 0.759 | 0.46 – 2.87 |
| Maternal postnatal only supplementation ≥400 IU/day (ref: <400 IU/d or none) | 0.95 | 0.923 | 0.36 – 2.54 |
| Maternal pre and postnatal supplementation ≥400 IU/day (ref: <400 IU/d or none) | 1.43 | 0.274 | 0.75 – 2.70 |
| Vaginal IAP (ref: vaginal no IAP) | **1.78** | **0.022** | **1.09 – 2.92** |
| CS-Elective (ref: vaginal no IAP) | **2.53** | **0.031** | **1.09 – 5.86** |
| CS-Emergency (ref: vaginal no IAP) | 1.78 | 0.070 | 0.95 – 3.31 |
| 2 cups milk per day (ref: ≤1 cup per day) | 0.56 | 0.136 | 0.26 – 1.20 |
| ≥3 cups milk per day (ref: ≤1 cup per day) | **0.40** | **0.012** | **0.19 – 0.82** |
| Age at stool sample collection (continuous) | 1.25 | 0.051 | 1.00 – 1.55 |
| Furry pets (ref: no furry pets) | **1.81** | **0.006** | **1.19 – 2.75** |
| Vancouver (ref: Edmonton) | **0.32** | **0.000** | **0.18 – 0.57** |
| Winnipeg (ref: Edmonton) | 0.60 | 0.096 | 0.33 – 1.09 |
| *^a^P values bolded if ≤0.05, calculated with logistic regression (adjusted for all other variables in the model)*  *Abbrev. aOR (adjusted odds ratio), CI (confidence interval)* | | | |
